# Ethyl Ferulate Suppresses Esophageal Squamous Cell Carcinoma Tumor Growth Through Inhibiting the mTOR Signaling Pathway

**DOI:** 10.3389/fonc.2021.780011

**Published:** 2022-01-28

**Authors:** Mengjun Pang, Xiaomeng Xie, Yuanyuan Zhang, Kyle Vaughn Laster, Kangdong Liu, Dong Joon Kim

**Affiliations:** ^1^ The Pathophysiology Department, The School of Basic Medical Sciences, Zhengzhou University, Zhengzhou, China; ^2^ China-US (Henan) Hormel Cancer Institute, Zhengzhou, China; ^3^ Translational Research Institute, Henan Provincial People’s Hospital, Academy of Medical Science, Zhengzhou University, Zhengzhou, China; ^4^ Provincial Cooperative Innovation Center for Cancer Chemoprevention, Zhengzhou University, Zhengzhou, China; ^5^ The Collaborative Innovation Center of Henan Province for Cancer Chemoprevention, Zhengzhou, China; ^6^ International Joint Research Center of Cancer Chemoprevention, Zhengzhou, China

**Keywords:** esophageal squamous cell carcinoma, mTOR, patient-derived xenograft, ethyl ferulate, cancer growth

## Abstract

Ethyl ferulate is a phenylpropanoid compound isolated from the medicinal herb *Ferula*. Although ethyl ferulate has anti-inflammatory, antioxidant, and neuroprotective activities with potential use in the nutraceutical and pharmaceutical industry, its anticancer effects and underlying molecular mechanisms against esophageal squamous cell carcinoma (ESCC) have not been investigated. This study investigates the anticancer activity and molecular mechanism of ethyl ferulate in ESCC. MTT, focus formation, soft agar, and cell cycle analysis were used to determine the effect of ethyl ferulate on cell proliferation and cell cycle. Potential candidate proteins were screened and verified *via* Western blotting, *in vitro* kinase assay, and *in vitro* pull-down assay. Mammalian target of rapamycin (mTOR) knockdown cell lines were established by lentiviral infection with shmTOR. The effect of ethyl ferulate on tumor growth was assessed using ESCC patient-derived xenograft models. Ethyl ferulate significantly inhibited cell growth and induced G1 phase cell cycle arrest in ESCC cells. Ethyl ferulate reduced the activity of mTOR *in vitro*. The inhibition of ESCC cell growth by ethyl ferulate is dependent on mTOR expression. In addition, ethyl ferulate strongly reduced ESCC patient-derived xenograft tumor growth in an *in vivo* mouse model. Ethyl ferulate is an mTOR inhibitor that can suppress ESCC progression and may be a novel candidate compound for esophageal cancer chemoprevention.

## Introduction

Esophageal cancer is one of the most common malignant digestive tract tumors worldwide, with a poor prognosis and high mortality (1). Esophageal squamous cell carcinoma (ESCC) accounts for approximately 90% of esophageal cancer cases (2). Treatment strategies for managing esophageal cancer have greatly improved; however, the 5-year survival rate of esophageal cancer patients is roughly 10%. Additionally, only 15%–40% of patients survive more than 5 years after post esophagectomy (3). Although epidermal growth factor receptor (EGFR) and vascular endothelial growth factor (VEGF) inhibitors have been used to treat ESCC therapeutically, patients initially benefiting from targeted therapies inevitably develop drug resistance (4, 5); therefore, it remains a worthwhile endeavor to investigate novel effective therapeutic targets and inhibitors to overcome these challenges.

The mammalian target of rapamycin (mTOR) is a serine/threonine kinase that drives cell growth and proliferation by linking a variety of extracellular signals and intracellular cues (6). In multicellular organisms, mTOR regulates cell growth and metabolism in response to growth factors, cellular energy conditions, and nutrients (7). The functions of mTOR are exerted *via* two different multicomponent complexes, mTORC1 and mTORC2, which cooperate with one another and several elements of other signaling pathways (8). The mTORC1 complex regulates protein synthesis and cell growth through the downstream molecules Eukaryotic initiation factor 4E-binding protein 1 (4E-BP1) and 70-kDa S6 kinase (p70S6K) (9). mTORC2 is responsive to growth factor signaling *via* phosphorylating downstream V-Akt murine thymoma viral oncogene homolog (AKT), Serum and glucocorticoid-regulated kinase (SGK); it also plays a vital role in the maintenance of normal and cancer cells by associating with ribosomes and participating in cellular metabolic regulation (10). AKT is a serine/threonine kinase and is a crucial Phosphoinositide 3-kinase (PI3K) signaling pathway component (11). The p70S6K is a serine/threonine protein kinase that plays an important role in cell growth, cell cycle, and cell differentiation (12). The p70S6K protein is an mTOR pathway effector, and the activation of the mTOR/p70S6K signaling pathway can stimulate protein synthesis and cell growth (13). mTOR is activated in a variety of malignant tumors and is an important regulator of cell proliferation and protein translation (14). mTOR might be a potential target strategy for ESCC treatment in the future because it is highly expressed in many human ESCC tumor tissues and is associated with various clinical characteristics (15–17). The deregulation of the mTOR signaling pathway in many cancers suggests that mTOR inhibitors may have broad therapeutic use in various tumor types (18). mTOR inhibitors have been used as monotherapy or combination therapy in preclinical and clinical trials to test its efficacy in treating various cancers; however, the effects were marginal, suggesting that the full therapeutic potential of targeting mTOR has not been exploited (19, 20). Thus, investigating more potent mTOR inhibitors for cancer treatment remains an ongoing challenge.

Ethyl ferulate, a monomeric phenylpropanoid compound isolated from the medicinal herb *Ferula*, has attracted extensive interest because of its anti-inflammatory activity, neuroprotective properties, and antioxidant activity (21, 22). Ethyl ferulate was found to prevent lipopolysaccharide (LPS)-induced acute lung injury (ALI) through regulating inflammatory responses (22). Ethyl ferulate was observed to ameliorate diabetes-induced oxidative stress and inflammation *via* activating nuclear factor erythroid 2-related factor 2 (Nrf2) pathway (23). Ethyl ferulate induced the level of heme oxygenase-1 (HO-1) expression and protected oxidative and neurodegenerative condition in brain cells (24). Additionally, ethyl ferulate prevented age-related macular degeneration through regulating the morphological and functional retinal degeneration (25). However, the anticancer activity of ethyl ferulate and its underlying molecular mechanisms against ESCC remain unclear. Herein, we investigated the anticancer effects of ethyl ferulate on the malignant growth of ESCC *in vivo* and *in vitro*.

## Materials and Methods

### Reagents and Antibodies

Reagents were purchased from the following companies: ethyl ferulate (purity >99% from BR analysis, Shanghai yuanye Bio-Technology Company, Shanghai, China), dimethyl sulfoxide (DMSO; Tianjin Kemai Chemical Reagent Company, Tianjin, China), Roswell Park Memorial Institute medium 1640 (RPMI1640) and fetal bovine serum (FBS; Biological Industries, Cromwell, CT, USA), Ham’s F12 medium (Lonza, Walkersville, MD, USA), and MEM/EBSS medium (GE Healthcare Life Sciences, Logan, UT, USA). The following antibodies were purchased from Cell Signaling Technology (Beverly, MA, USA) for performing Western blot assays: phosphorylated mTOR (S2481; Cat# 2974), phosphorylated P70S6K (T389; Cat# 9234), phosphorylated AKT (S473; Cat# 4060), phosphorylated GSK3β (S9; Cat# 5558), phosphorylated ERK (T202/Y204; Cat# 4370), total mTOR (Cat# 2972), total P70S6K (Cat# 2708), total AKT (Cat# 4691), total GSK3β (Cat# 12456), total ERK (Cat# 4695), Cyclin D1 (Cat# 2922), p21 (Cat# 2947), and Ki-67 (Cat# 9027). The antibody for β-actin detection was purchased from Santa Cruz Biotechnology (Santa Cruz, CA, USA), and the horseradish peroxidase (HRP)-conjugated secondary antibodies were purchased from ZSGB-BIO (Beijing, China). Active mTOR recombinant protein used for *in vitro* kinase assay or *in vitro* pull-down assay was purchased from Thermo Fisher (Shanghai, China), and inactive p70S6K recombinant protein used for *in vitro* kinase assay was purchased from SignalChem (Richmond, BC, Canada).

### Cell Culture

ESCC cell lines (KYSE30, KYSE70, KYSE450, and KYSE510) and JB6 mouse epithelial cells were purchased from the Chinese Academy of Sciences (Shanghai, China) and American Type Culture Collection (ATCC) (Manassas, VA, USA), respectively. SHEE normal esophageal cells were provided by Dr. Yan Zheng (The Affiliated Cancer Hospital, Zhengzhou, China). All cell lines were cytogenetically validated before the experiments. Each cell line was thawed and maintained in culture medium up to 8 weeks. The KYSE30 cells were cultured in a 1:1 mixture of RPMI 1640 medium and Ham’s F12 medium supplemented with 2% FBS. RPMI1640 medium supplemented with 10% FBS was used to culture the other ESCC cells. JB6 mouse epithelial cells were cultured in MEM/EBSS medium supplemented with 5% FBS. All media were supplemented with 1% penicillin/streptomycin. All cells were maintained at 37°C in a humidified incubator under 5% CO_2_.

### MTT Assay

KYSE30 (2.5 × 10^3^ cells per well), KYSE70, KYSE450, or KYSE510 (2.0 × 10^3^ cells per well) cells suspended in complete growth medium were seeded in 96-well plates (100 μl/well). After overnight incubation, different concentrations of ethyl ferulate or isometric DMSO were diluted in complete growth medium and added to each well (100 μl/well). After 72 h, 20 μl of the MTT solution (Solarbio, Beijing, China) were added to each well and the cells were incubated for 2 h at 37°C. Next, the cell culture medium was removed and 150 μl of DMSO were added to each well. The formazan crystals were dissolved by gentle agitation, and cell proliferation was measured at 570 nm absorbance using the Thermo Multiskan plate-reader (Thermo Fisher Scientific, Waltham, MA, USA).

### Anchorage-Independent Cell Growth Assay

KYSE450 or KYSE510 cells (8 × 10^3^ cells per well) were suspended in 0.3% agar medium (RPMI 1640 containing various concentrations of ethyl ferulate) and then plated on a 0.6% agar containing the same concentrations of ethyl ferulate in 6-well plates. Cells were maintained at 37°C in a humidified incubator under 5% CO_2_ for 2 weeks. Colonies were then photographed with an inverted microscope and counted using the Image-Pro Plus software (v.6) program (Media Cybernetics, Rockville, MD, USA).

### Focus Formation Assay

KYSE450 or KYSE510 cells (1 × 10^3^ cells per well) were seeded into 6-well plates and incubated overnight before treatment with various concentrations of ethyl ferulate. Cells were maintained at 37°C in a humidified incubator under 5% CO_2_ for 10 days. Foci were stained by 0.4% crystal violet staining solution, and the number of foci was counted.

### Cell Cycle Analysis

KYSE30, KYSE450, or KYSE510 cells were seeded (9 × 10^4^ cells per 60-mm dish) and incubated for 24 h. The cells were then treated with various concentrations of ethyl ferulate or DMSO for 48 h. The cells were then collected by trypsinization, washed with 1× PBS, and fixed in 70% ethanol at -20°C overnight. After rehydration, cells were incubated in RNase (10 mg/ml, Solarbio) for 1 h and then stained with propidium iodide (PI; 20 μg/ml, Solarbio). The cell cycle was analyzed by flow cytometry.

### *In Vitro* Kinase Assay

The kinase assay was performed according to the manufacturer’s instructions (Promega, Madison, WI, USA). Briefly, the active recombinant mTOR (50 ng) protein was mixed with different concentrations of ethyl ferulate in reaction buffer and incubated at room temperature for 15 min. Next, the inactive p70S6K recombinant protein (100 ng) and ATP (Cell Signaling Technology) were added and incubated for 30 min at 30°C. The reaction was stopped by adding 10 μl protein loading buffer. The protein band was detected by Western blot analysis. mTOR activity was assessed using a p70S6K phosphorylation antibody.

### *In Vitro* Pull-Down Assay

Recombinant mTOR (200 ng) protein or ESCC cell lysate from KYSE510 (1 mg) was incubated with ethyl ferulate–Sepharose 4B or Sepharose 4B beads (100 μl, 50% slurry) in reaction buffer. Incubated samples were constantly rotated at 4°C overnight. The following day, the beads were washed 5 times and the protein bands were detected by Western blot analysis.

### Establishing mTOR Knockdown Cells

Small hairpin RNA plasmids against mTOR were constructed as previously described (26). Viral vectors were co-transfected with packaging vectors into Lentix-293T cells using Lipofectamine 2000 (Invitrogen, Grand Island, NY, USA) to generate lentivirus. At 48 h after transfection, the virus-enriched medium was harvested and passed through a 0.45-μm filter. The virus-enriched medium was then supplemented with 10 μg/ml of polybrene and added to KYSE450 and KYSE510 cells grown to ~60% confluence. The cells were incubated overnight. The following day, the cell culture medium was replaced with fresh complete medium. Infected cells were then selected with puromycin (1 μg/ml) for 48 h.

### *In Vivo* Patient-Derived Esophageal Squamous Cell Carcinoma Xenograft Model and Ethics Statement

The study was conducted according to guidelines established for the care and use of laboratory mice by the Zhengzhou University Institutional Animal Care and Use Committee (Zhengzhou, China). Severe combined immunodeficiency (SCID) female mice (6–9 weeks old) were used to examine the effect of ethyl ferulate on ESCC patient-derived tumor growth. Human ESCC tumor tissues were excised from Affiliated Cancer Hospital in Zhengzhou University patients who received neither chemotherapy nor radiotherapy treatments before surgery. Tissue histology was confirmed by a pathologist. Informed consent was given by each patient. After tumors grew to an average volume of approximately 200 mm^3^, the mice were randomly divided into 2 groups containing 20 animals of the LEG110 tissue and 14 animals of the LEG45 tissue as follows: 1) untreated vehicle group (n = 10 or 7, respectively) and 2) 100 mg ethyl ferulate/kg of body weight (n = 10 or 7, respectively). Ethyl ferulate or vehicle (20% DMSO in 20% Tween 80) was administered by oral gavage once per day Monday through Friday. Tumor volumes were measured by Vernier caliper and calculated according to the following formula: tumor volume (mm^3^) = (length × width × height × 0.52). Mice were monitored until the tumor volume reached approximately 1.5 cm^3^. The mice were then euthanized, and their tumors, blood, livers, kidneys, and spleens were extracted.

### Hematoxylin–Eosin Staining and Immunohistochemistry

The liver, spleen, kidney, and tumor tissues from mice were prepared for hematoxylin–eosin (H&E) staining or immunohistochemistry (IHC). For H&E staining, the tissue sections were deparaffinized, hydrated, stained with H&E, and then dehydrated. For IHC, tumor tissue sections were baked at 65°C and hydrated. Afterward, the tissue sections were boiled in sodium citrate buffer (10 mM, pH 6.0) for antigen retrieval and then treated with H_2_O_2_ for 5 min. After incubation with a primary antibody (Ki-67, 1:100) at 4°C in a humidified chamber overnight, the sections were incubated with an HRP-conjugated goat anti-rabbit or mouse IgG antibody (ZSGB-BIO, Beijing, China) for 30 min. Tissue sections were then stained with 3,3′-diaminobenzidine (DAB; ZSGB-BIO, Beijing, China). The IHC staining was observed by microscope and quantified using the Image-Pro Plus software program.

### Statistical Analysis

All statistical results were presented as mean values ± SD. The Student’s *t* test or one-way analysis of variance (ANOVA) was used to compare significant differences. Statistical analyses were performed using Statistical Package for the Social Sciences (SPSS) for Windows (IBM, Inc., Armonk, NY, USA). Statistical significance was considered at *P* value <0.05.

## Results

### Ethyl Ferulate Suppresses Esophageal Squamous Cell Carcinoma Cell Growth

**Figure 1:**
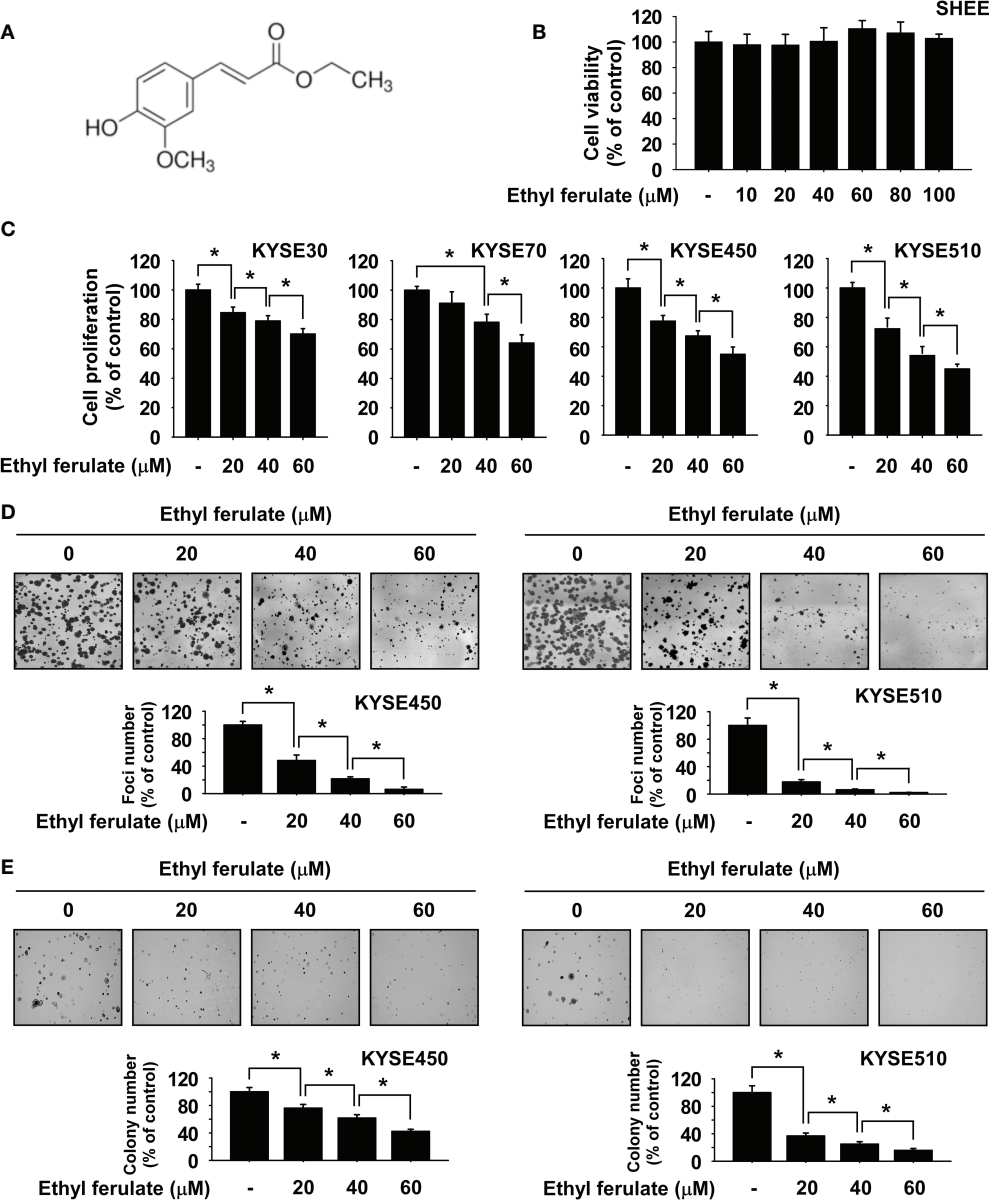
Ethyl ferulate inhibits esophageal squamous cell carcinoma (ESCC) cell growth. **(A)** Chemical structure of ethyl ferulate. **(B)** The cytotoxicity of ethyl ferulate was determined using SHEE normal esophageal cells. Cell growth was determined by MTT assay. **(C)** Effect on the growth of ESCC cells by ethyl ferulate treatment. Cell growth was determined by MTT assay. **(D)** Effect on focus formation upon treatment with ethyl ferulate. Focus ability was determined by the focus formation assay. **(E)** Effect on anchorage-independent ESCC cell growth upon treatment with ethyl ferulate. Colonies were imaged using a microscope and quantified with the Image-Pro PLUS (v.6) computer software program. For panels **(B–E)**, data are shown as means ± SD of values from 3 independent experiments each with triplicate samples. The asterisk (*) indicates a significant (*P* < 0.05) inhibitory effect of ethyl ferulate.

Ethyl ferulate is a 4-hydroxy-3-methoxycinnamate compound (**Figure 1A**). SHEE normal esophageal cells were used to assess the toxicity of ethyl ferulate. Cell viability was determined by MTT assay after cells were treated with several concentrations of ethyl ferulate for 72 h. Results showed that SHEE normal esophageal cell proliferation was not affected by ethyl ferulate treatment (**Figure 1B**). Therefore, we used ethyl ferulate at concentrations of 20, 40, and 60 μM for the subsequent experiments. To assess the effect of ethyl ferulate on ESCC cell proliferation, we next treated KYSE30, KYSE70, KYSE450, or KYSE510 ESCC cells with ethyl ferulate at different concentrations for 72 h. The MTT assay results revealed that treatment with ethyl ferulate significantly inhibited the growth of ESCC cells in a dose-dependent manner (**Figure 1C**). Furthermore, the focus formation and anchorage-independent ESCC cell growth assays showed that ethyl ferulate suppressed focus number and anchorage-independent growth of KYSE450 and KYSE510 ESCC cells in a dose-dependent manner (**Figures 1D, E**).

### Ethyl Ferulate Increases G1 Phase Cell Cycle Arrest in Esophageal Squamous Cell Carcinoma Cells

**Figure 2:**
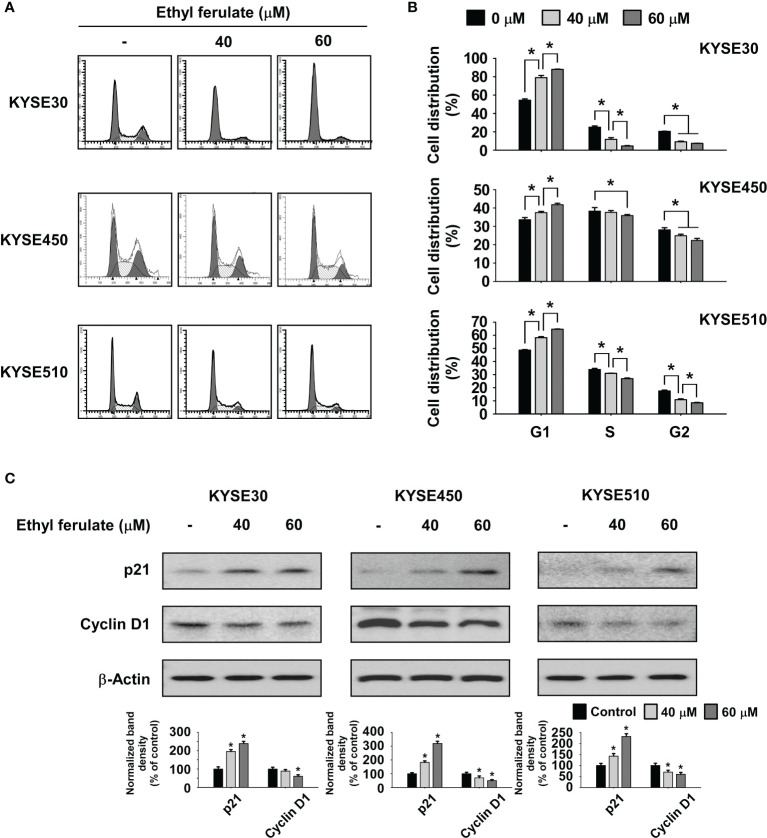
Ethyl ferulate induces G1 phase cell cycle arrest. **(A, B)** The effect of ethyl ferulate on the cell cycle in KYSE30, KYSE450, or KYSE510 cells was analyzed by fluorescence-activated cell sorting (FACS). Data are indicated as means ± SD of values from 3 independent experiments, and the asterisk (*) indicates a significant (*P* < 0.05) difference between ethyl ferulate-treated cells and dimethyl sulfoxide (DMSO)-treated cells. **(C)** The effect of ethyl ferulate on the expression of cell cycle marker proteins was determined by Western blotting. Band density was measured using the ImageJ (NIH) software program. For panel **(C)**, similar results were observed from 3 independent experiments and band density is shown as a bar graph. One-way ANOVA was used to compare significant differences.

Flow cytometry analysis was performed to determine whether treatment with ethyl ferulate affected cell cycle regulation in ESCC cell lines. KYSE30, KYSE450, or KYSE510 ESCC cells were treated with ethyl ferulate at several concentrations for 48 h. The results indicated that ethyl ferulate induced G1 phase cell cycle arrest in a dose-dependent manner (**Figures 2A, B**). Based on the perturbation of the cell cycle induced by ethyl ferulate, we determined the expression of cell cycle marker proteins in KYSE30, KYSE450, and KYSE510 cells after ethyl ferulate treatment. KYSE30, KYSE450, or KYSE510 ESCC cells were treated with ethyl ferulate for 48 h, and cell cycle marker proteins were analyzed by Western blotting. The results of Western blotting indicated that ethyl ferulate increased the expression of p21 and decreased the expression of cyclin D1 (**Figure 2C**).

### Ethyl Ferulate Is a Novel mTOR Inhibitor

**Figure 3:**
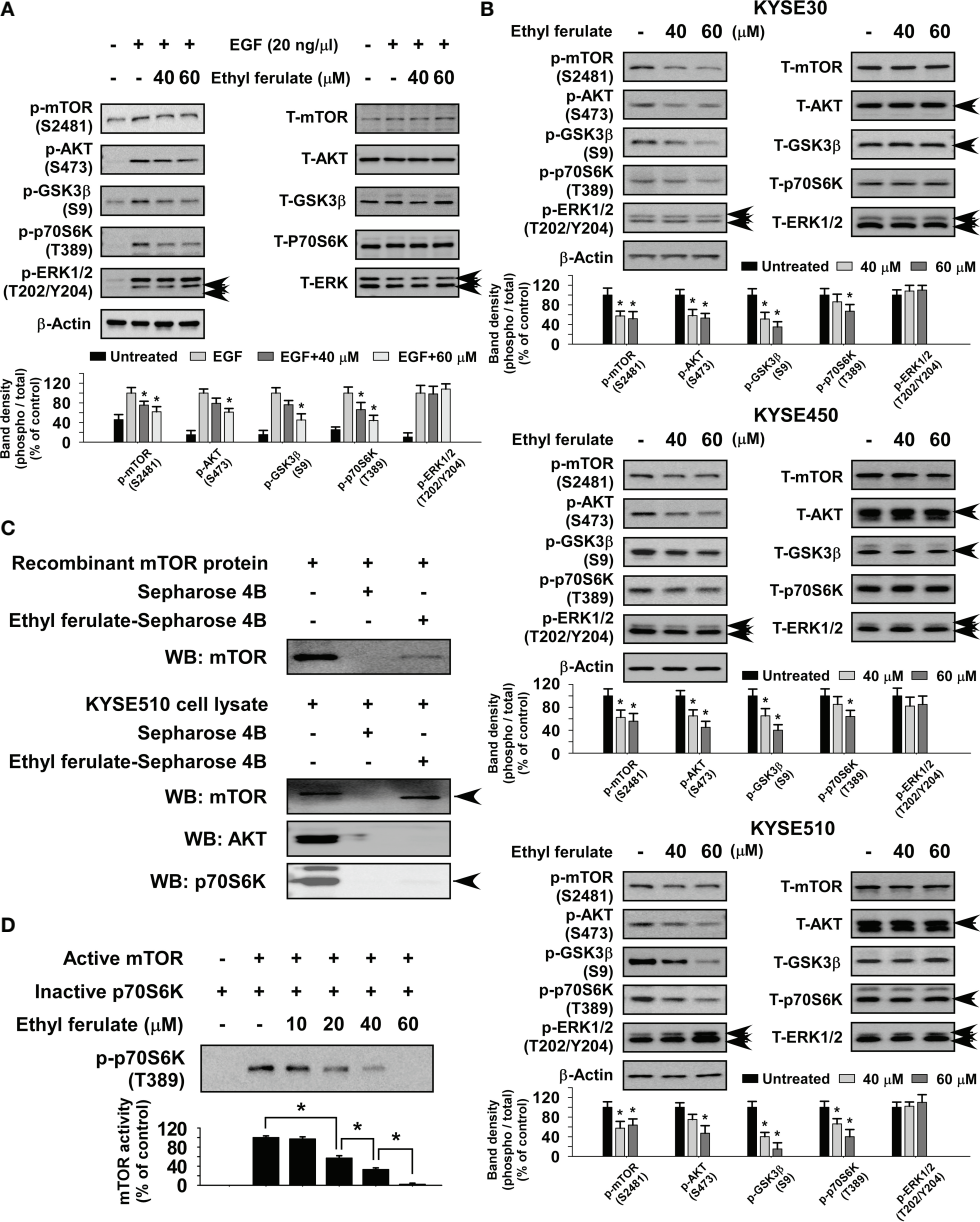
Ethyl ferulate strongly inhibits the mammalian target of rapamycin (mTOR) signaling pathway. **(A)** The effect of ethyl ferulate on epidermal growth factor (EGF)-induced kinase signaling in JB6 cells. Serum-starved [without fetal bovine serum (FBS); 24 h] cells were treated with different concentrations of ethyl ferulate for 6 h followed by treatment with EGF for 30 min. **(B)** The effect of ethyl ferulate on various kinase signaling proteins in esophageal squamous cell carcinoma (ESCC) cells. Cells were treated with ethyl ferulate for 24 h, and then various signaling proteins were examined by Western blotting. **(C)** Ethyl ferulate directly binds to recombinant mTOR protein and mTOR present in KYSE510 cell lysates. The recombinant mTOR protein or cell lysate was incubated with Sepharose 4B beads or ethyl ferulate-conjugated Sepharose 4B beads. Pulled down proteins were examined by Western blotting. **(D)** The effect of ethyl ferulate on mTOR kinase activity. mTOR kinase activity was assessed by *in vitro* kinase assay using active mTOR and inactive p70S6K proteins. For all experiments, similar results were shown as mean values ± SD for 3 independent experiments and band density is shown as a bar graph. One-way analysis of variance (ANOVA) was used to analyze the data. The asterisk (*) indicates a significant (*P* < 0.05) inhibitory effect of ethyl ferulate.

To determine the underlying molecular mechanism induced upon treatment with ethyl ferulate, we first investigated whether it could affect various EGF-induced signaling molecules in JB6 cells. After 24-h serum starvation, cells were treated with ethyl ferulate for 6 h and then subsequently treated with EGF for 0.5 h. The results indicated that the phosphorylation of mTOR, AKT, GSK3β, and p70S6K was strongly decreased by treatment with ethyl ferulate in a dose-dependent manner, whereas the protein level of phosphorylated ERK1/2 was not affected (**Figure 3A**). Therefore, to further identify the molecular targets of ethyl ferulate, we evaluated the effect of ethyl ferulate on signaling pathways in KYSE30, KYSE450, and KYSE510 cells after treatment with ethyl ferulate for 24 h. Phosphorylated mTOR, AKT, GSK3β, and p70S6K protein levels were strongly reduced after treatment with ethyl ferulate; however, the level of phosphorylated ERK1/2 was unaffected (**Figure 3B**). To determine potential targets of ethyl ferulate, we performed *in vitro* pull-down assays using ethyl ferulate-conjugated Sepharose 4B beads, Sepharose 4B with a recombinant mTOR protein, or KYSE510 cell lysates. The results showed that ethyl ferulate directly binds with mTOR but not AKT or p70S6K proteins (**Figure 3C**). Furthermore, we performed *in vitro* kinase assays using an active recombinant mTOR protein and an inactive p70S6K protein to confirm the effect of ethyl ferulate on mTOR activity. The result indicated that ethyl ferulate suppressed the phosphorylation of p70S6K by directly targeting mTOR in a dose-dependent manner (**Figure 3D**). Additionally, to determine whether the activities of other signaling proteins were affected by ethyl ferulate, the activities of a panel of 36 recombinant active kinases and their respective substrates were screened in the presence or absence of ethyl ferulate. The results indicated that mTOR was the sole kinase that was strongly inhibited by ethyl ferulate (**Supplementary Figures S1A, B**).

### Knockdown of mTOR Suppresses Esophageal Squamous Cell Carcinoma Cell Growth

**Figure 4:**
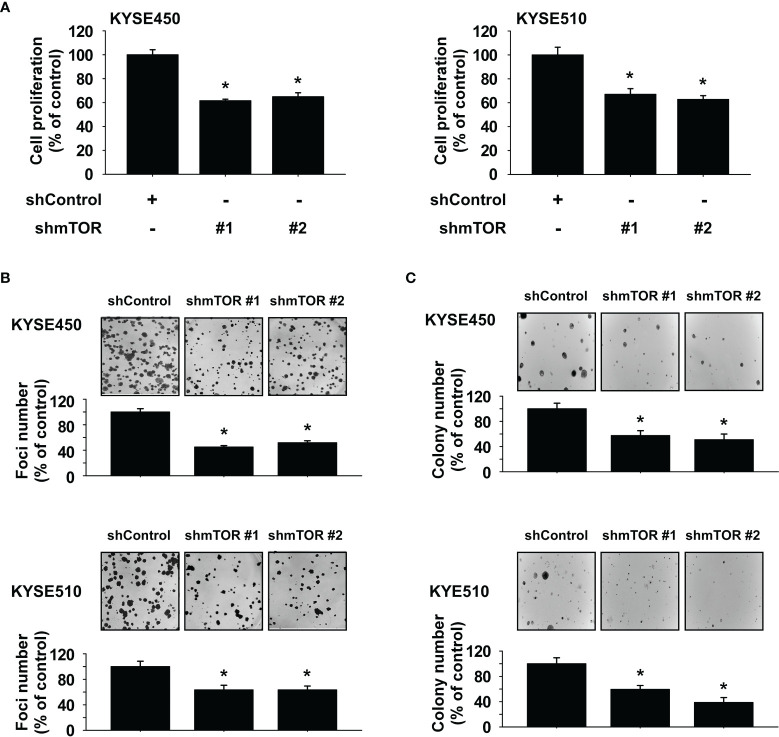
Mammalian target of rapamycin (mTOR) is a therapeutic target in esophageal squamous cell carcinoma (ESCC) cells. **(A)** Effect of mTOR knockdown (shmTOR) on ESCC cell growth was determined by MTT assay. **(B)** Effect of mTOR knockdown (shmTOR) on focus formation ability of ESCC cells. Cells were incubated for 10 days, and then focus number was counted. **(C)** Effect on anchorage-independent growth of ESCC cells by mTOR knockdown (shmTOR). After incubation for 2 weeks, colony number was counted. For all, data are shown as means ± SD of values from 3 independent experiments, and the asterisk (*) indicates a significant (*P* < 0.05) inhibitory effect of mTOR knockdown (shmTOR). One-way analysis of variance (ANOVA) with Dunnett’s *post-hoc* test was used to compare significant differences.

To determine the influence of mTOR knockdown on ESCC cell growth, we established stable shRNA control or shmTOR cell lines and subsequently used Western blotting to detect the expression level of mTOR protein. The expression of phosphorylated and total mTOR was strongly suppressed in shmTOR #1 and shmTOR #2 cells (**Supplementary Figures S2A, B**). We next examined the effect of mTOR knockdown on ESCC cell growth using an MTT assay. The results indicated that the anchorage-dependent cell growth of KYSE450 and KYSE510 ESCC cells was decreased upon knockdown of mTOR (**Figure 4A**). Furthermore, the focus formation and soft agar assay results showed that focus number and anchorage-independent growth of ESCC cells were significantly reduced upon knockdown of mTOR (**Figures 4B, C**).

### The Inhibition of Esophageal Squamous Cell Carcinoma Cell Growth by Ethyl Ferulate Is Dependent on mTOR Signaling

**Figure 5:**
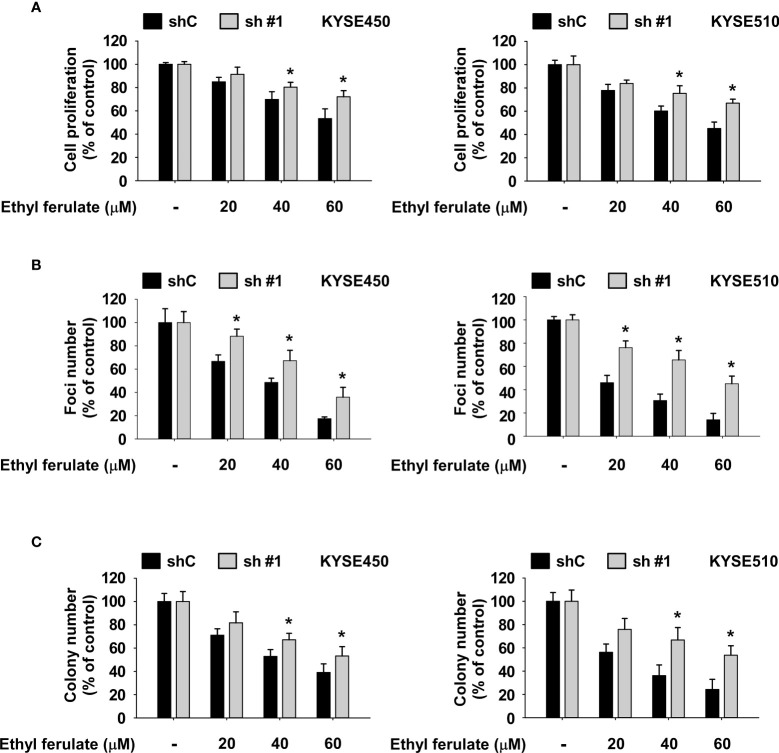
The inhibition of esophageal squamous cell carcinoma (ESCC) cell growth by ethyl ferulate is dependent on the expression of mammalian target of rapamycin (mTOR). **(A, B)** The effect of ethyl ferulate on ESCC cancer cell growth and focus formation ability was assessed in cells stably expressing shmTOR or cells stably expressing shControl. Cells were treated with or without ethyl ferulate for 72 h or 10 days, respectively. Cell growth was analyzed by MTT and focus formation assays. **(C)** The effect of ethyl ferulate on anchorage-independent ESCC cell growth was assessed in cells stably expressing shmTOR or cells stably expressing shControl. Cells were treated with or without ethyl ferulate for 2 weeks, and then colonies were counted using a microscope and the Image-Pro PLUS (v.6) computer software program. All data are shown as means ± SD of values from 3 independent experiments. The asterisk (*) indicates a significant effect (*P* < 0.05) of ethyl ferulate treatment between mTOR knockdown cells and shControl cells. The Student’s *t* test with one-way ANOVA was used to compare significant differences.

We next assessed whether the effect of ethyl ferulate on ESCC cell proliferation is dependent on the expression of mTOR. shmTOR cells were treated with ethyl ferulate for 72 h, and then anchorage-dependent growth was assessed by MTT assay. Results indicated that shmTOR #1 cells were resistant to the growth inhibitory effect of ethyl ferulate compared with shControl cells (**Figure 5A**). Additionally, the focus formation assay and soft agar assay results showed that the inhibitory effect of ethyl ferulate in shmTOR cells is reduced relative to shControl growth (**Figures 5B, C**).

### Ethyl Ferulate Inhibits Esophageal Squamous Cell Carcinoma Tumor Growth *In Vivo*

**Figure 6:**
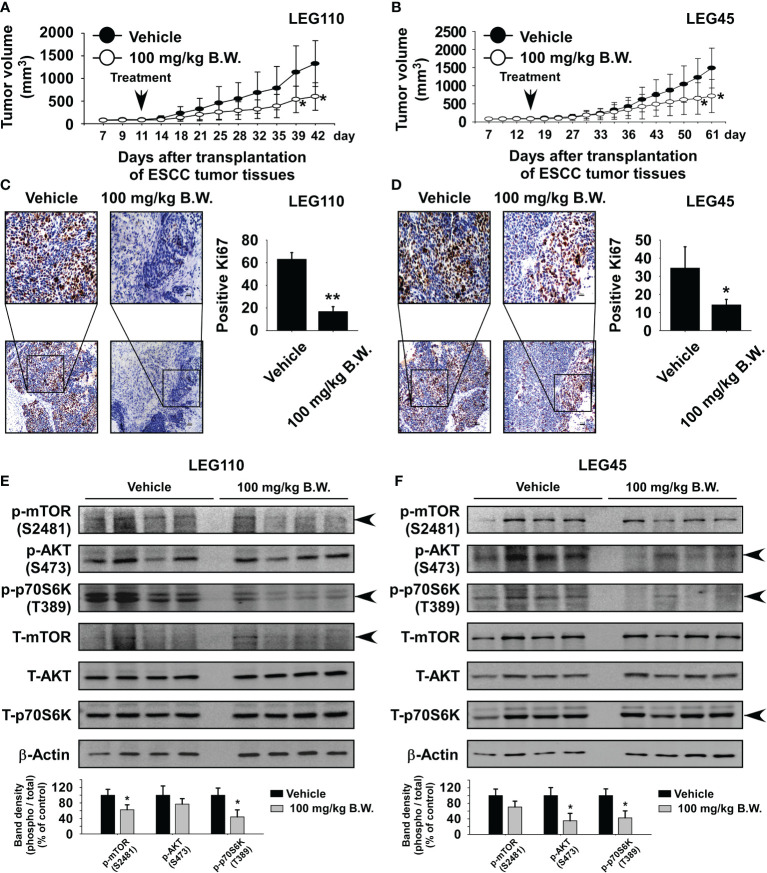
Ethyl ferulate reduces esophageal squamous cell carcinoma (ESCC) patient-derived xenograft tumor growth *in vivo.* Mice were divided into two groups to assess the effect of ethyl ferulate on ESCC patient-derived xenograft (PDX) tumor growth. Groups are as follows: 1) vehicle group or 2) group treated with 100 mg/kg of ethyl ferulate. Tumor-bearing mice were orally treated (by gavage) with ethyl ferulate or vehicle for 32 (LEG110; n = 10) or 53 (LEG45; n = 7) days. Tumor volumes were measured on the days indicated. The effect of ethyl ferulate on ESCC tumor growth in **(A)** LEG110 or **(B)** LEG45 ESCC PDX tissues. For panels **(A, B)**, data are shown as mean ± SE from each group. The effect of ethyl ferulate on Ki-67 expression in **(C)** LEG110 or **(D)** LEG45 ESCC PDX tissues (n = 5). Three slices per PDX tissue were analyzed. Treated or untreated groups of tumor tissues were stained with antibodies to detect Ki-67 (×100; scale bar: 100 μm; ×20; scale bar: 50 μm). **(E, F)** Ethyl ferulate inhibits phosphorylated mammalian target of rapamycin (mTOR), p70S6K, and AKT protein expression in esophageal tumor tissues. Tumor tissues from each group were immunoblotted with antibodies to detect mTOR, p70S6K, and AKT and phosphorylated mTOR, p70S6K, AKT, and β-actin. β-Actin was used to verify equal protein loading. Band density was measured using the ImageJ (NIH) software program, and the results are shown as a bar graph. One-way ANOVA was used to compare significant differences. The asterisk (*) indicates a significant (*P < 0.05; **P < 0.01) inhibitory effect of ethyl ferulate treatment.

We first assessed the toxicity profile of ethyl ferulate to determine the appropriate concentration for use in our *in vivo* studies. Mice were orally administered vehicle, ethyl ferulate at 50 mg/kg, or ethyl ferulate at 100 mg/kg once a day Monday through Friday for 2 weeks by the gavage method. Blood samples from mice were collected and used to analyze alanine aminotransferase (ALT), aspartate aminotransferase (AST) activity. The results showed that ALT and AST activities were not significantly altered in mice treated with ethyl ferulate at 50 or 100 mg/kg compared with the vehicle-treated group (**Supplementary Figures S3A, B**; n = 4). Therefore, we used 100 mg/kg ethyl ferulate for the ESCC patient-derived xenograft (PDX) study. To evaluate the antitumor activity of ethyl ferulate *in vivo*, we used two PDX models: LEG110 and LEG45. LEG110 or LEG45 human ESCC tumor tissues were implanted into the back of the necks of SCID mice, and ethyl ferulate at 100 mg/kg or vehicle was administered by gavage once a day for 32 (LEG110 tissue; n = 10) or 53 (LEG45 tissue; n = 7) days. The results indicated that the treatment of mice with ethyl ferulate significantly suppressed the tumor volume relative to the vehicle-treated group (**Figures 6A, B**). Additionally, mice that were treated with ethyl ferulate exhibited no significant loss of body weight compared to the vehicle-treated group (**Supplementary Figures S4A, B**). Next, we measured the AST and ALT activity from serum to determine the potential toxicity of ethyl ferulate on PDX mice. Results showed that the ALT and AST activities were not significantly altered in mice treated with ethyl ferulate compared with the vehicle-treated group (Supplementary Figure S4C). We next prepared the tumor tissues that were isolated from both PDX cases for IHC staining of Ki-67, a cell proliferation marker protein. Results indicated that the expression of Ki-67 was significantly decreased in LEG110 or LEG45 tissues by ethyl ferulate treatment compared to the vehicle-treated group (**Figures 6C, D**; n = 5). Furthermore, the liver, kidney, and spleen tissues were stained with H&E to evaluate the potential toxicity of ethyl ferulate. The results of the H&E staining showed that there were no obvious morphological changes between treated and untreated groups (**Supplementary Figures S5A, B**; n = 5). Next, Western blotting was performed to investigate whether ethyl ferulate could inhibit protein expression of mTOR and its downstream signaling targets in PDX tumor tissues. The results showed that the phosphorylation of mTOR, AKT, and p70S6K was strongly inhibited in the ethyl ferulate-treated group (**Figures 6E, F**). We could suggest that inhibition of mTOR activity by ethyl ferulate exerts multiple effects on tumor progression, cancer growth, and cell cycle by modulating the mTOR signaling pathway (**Supplementary Figure S6**).

## Discussion

Despite comprehensive molecular characterization of mTOR, available targeted therapies for ESCC are still lagging behind (27). Ethyl ferulate is a phenylpropanoid compound that has been reported to have potential uses in the nutraceutical and pharmaceutical industry (28). However, the potential therapeutic effects of ethyl ferulate and its molecular targets have not been investigated in cancer. In the present study, we report that ethyl ferulate suppresses ESCC growth by targeting mTOR *in vitro* and *in vivo*.

Ribosomal protein S6 kinase (P70S6K) is the downstream effector of mTORC1, and AKT is a key substrate of mTORC2 (29, 30). mTORC1 is sensitive to nutrients, and mTORC2 is regulated through PI3K and growth factor signaling. Both complexes influence each other, as AKT regulates PRAS40 phosphorylation to facilitate mTORC1 activity, while p70S6K regulates Sin1 to modulate mTORC2 activity (31). Through the results of target screening, *in vitro* pull-down assays, and *in vitro* kinase assays, we identified that ethyl ferulate is a potent mTORC1/mTORC2 protein kinase inhibitor that can reduce mTOR/AKT/p70S6K signaling in ESCC cells (**Figures 3A, B**).

Ethyl ferulate directly bound mTOR protein (**Figure 3C**) and suppressed the activity of mTOR protein kinase (**Figure 3D**). Additionally, shmTOR cells were resistant to anticancer effects of ethyl ferulate (**Figures 5A**–**C**). However, high concentrations of ethyl ferulate still reduced cell growth in mTOR knockdown cells (**Figures 5A**–**C**). Therefore, ethyl ferulate may have additional molecular targets. To determine other molecular targets of ethyl ferulate, we conducted a cancer-related kinase screening. However, we did not identify other targets within the kinase panel (**Supplementary Figures S1A, B**). Therefore, we will investigate additional potential target proteins of ethyl ferulate in the future.

As reported, the disappointing performance of investigational anticancer candidates implies that there are some shortcomings in the translation of preclinical *in vitro* and *in vivo* models to humans and that heterogeneity in the patient population presents a significant challenge (32). However, PDX models have become the most credible *in vivo* human cancer model, as the primary patient tumor characteristics, including gene expression profiles and drug responses, are retained (33). Therefore, the PDX model is useful for drug screening, biomarker development, and the preclinical evaluation of personalized medicine (34). In this study, we first reported the anticancer activity of ethyl ferulate using ESCC PDX models. The results indicated that ethyl ferulate treatment reduced patient-derived esophageal xenograft tumor growth by inhibiting the mTOR/AKT/p70S6K signaling pathway (**Figures 6A, B, E, F**). Therefore, targeting mTOR protein with ethyl ferulate may provide antineoplastic effects against esophageal cancer.

## Data Availability Statement

The raw data supporting the conclusions of this article will be made available by the authors, without undue reservation, to any qualified researcher.

## Ethics Statement

All experiments involving animals were performed with the permission and under the strict guidance of the Zhengzhou University Institutional Animal Care and Use Committee (Zhengzhou, Henan, China).

## Author Contributions

MP: writing—original draft preparation, validation, and formal analysis. XX and YZ: methodology and formal analysis. KVL and KDL: writing—editing and investigation. DK: supervision, project administration, and funding.

## Funding

This work was supported by Henan Joint Fund, China (grant number U1804196); General project, National Natural Science Foundation China (NSFC) (grant numbers 81872335, 82103193); and Youth Science Foundation of Natural Science Foundation of Henan Province, China (grant number 212300410315).

## Conflict of Interest

The authors declare that the research was conducted in the absence of any commercial or financial relationships that could be construed as a potential conflict of interest.

## Publisher’s Note

All claims expressed in this article are solely those of the authors and do not necessarily represent those of their affiliated organizations, or those of the publisher, the editors and the reviewers. Any product that may be evaluated in this article, or claim that may be made by its manufacturer, is not guaranteed or endorsed by the publisher.

## Supplementary Material

Supplementary Figure 1 Effect of Ethyl ferulate on the activity of various kinases. **(A, B)** ABL, AKT1, AMPKα1, AURUA, B-RAF, CDK2/Cyclin E, CDK4/Cyclin D3, CHEK1, DNA-PK, EGFR, ERBB2, ERK1, FAK, FGFR1, FYN, GSK3β, HIPK1, IKKα, JAK1, KIT, MEK1, MET, MKK6, MSK1, mTOR/FKBP12, NEK1, p70S6K, PAK4, PDK1, PI3K(p110α/p65α), PIM1, PKCα, RSK2, SRC, TBK1, or TLK1 kinase and their respective substrates were incubated with or without Ethyl ferulate in an in vitro kinase assay. Data are shown as mean ± S.D of values.

Supplementary Figure 2 Expression of mTOR protein in mTOR knockdown cells. **(A, B)** Effect of mTOR knockdown on total or phosphorylated mTOR protein expression in KYSE450 **(A)** and KYSE510 **(B)** cells. KYSE450 and KYSE510 cells stably expressing shRNA control or shmTOR were analyzed by Western blotting. Band density was measured using the Image J (NIH) software program. For all experiments, similar results were observed from 3 independent experiments and band density is shown as a bar graph. One-way ANOVA was used to compare significant differences.

Supplementary Figure 3 Effect of Ethyl ferulate on ALT and AST activity. **(A, B)** The effect of Ethyl ferulate on ALT **(A)** and AST activity **(B)** was determined. Mice were orally administrated Ethyl ferulate at 50 mg/kg, 100 mg/kg, or vehicle once a day Monday through Friday for 2 weeks by the gavage method. Blood samples from mice were collected and analyzed. The ALT and AST activity were measured in mice treated with Ethyl ferulate and the vehicle-treated group. Data are shown as mean ± S.E. of values obtained from each group (n = 4).

Supplementary Figure 4 Effect of Ethyl ferulate on mice bodyweight, ALT and AST activity. **(A, B)** Effect of Ethyl ferulate on mice body weight. Vehicle or Ethyl ferulate at 100mg/kg was administered to mice by oral gavage, once a day for 32 (LEG110 tissue) or 53 (LEG45 tissue) days. The body weights of mice were treated with Ethyl ferulate or vehicle were measured once a week over the timespan of 32 (LEG110 tissue) or 53 (LEG45 tissue) days. **(C)** Ethyl ferulate exhibits no obvious toxicity *in vivo*. The effect of Ethyl ferulate on the ALT and AST activity were determined. Blood samples from each group (n = 7) were collected and analyzed. The ALT and AST activity were measured in mice treated with Ethyl ferulate or vehicle. All data are shown as mean ± S.E. of values obtained from experiments.

Supplementary Figure 5 Ethyl ferulate has no obvious toxicity in vivo. **(A, B)** H&E staining of liver, kidney, or spleen isolated from Ethyl ferulate-treated and untreated mouse groups inoculated with LEG110 (A; n = 5, 3 slides per tissue) and LEG45 (B; n = 5, 3 slides per tissue) tissues (100×; Scale bar: 100 μm).

Supplementary Figure 6 Representative diagram of the mechanism of the anti-cancer activity of Ethyl ferulate.

## Abbreviations

AKT, V-Akt murine thymoma viral oncogene homolog; mTOR, mammalian target of rapamycin; p70S6K, p70 ribosomal S6 kinase; PDX, patient-derived xenograft; RAPTOR, regulatory-associated protein of mTOR; RICTOR, rapamycin-insensitive companion of mTOR.
