# Supplementary figures and images for: Ethyl Ferulate Suppresses Esophageal Squamous Cell Carcinoma Tumor Growth Through Inhibiting the mTOR Signaling Pathway
Source: Front Oncol. 2022 Jan 28;11:780011. doi: 10.3389/fonc.2021.780011 (PMC8833257; doi:10.3389/fonc.2021.780011)

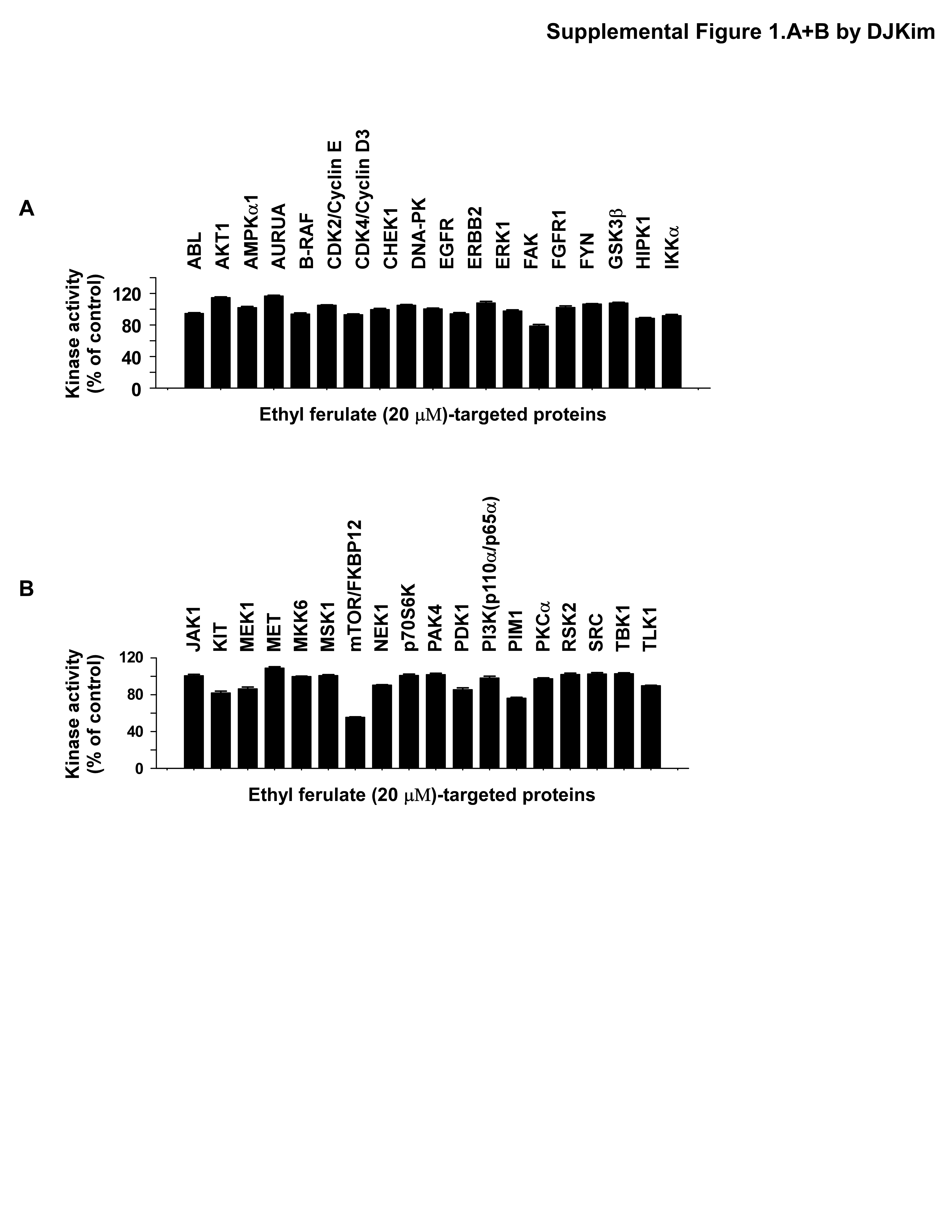

Supplement: Supplementary Figure 1 — Effect of Ethyl ferulate on the activity of various kinases. (A, B) ABL, AKT1, AMPKα1, AURUA, B-RAF, CDK2/Cyclin E, CDK4/Cyclin D3, CHEK1, DNA-PK, EGFR, ERBB2, ERK1, FAK, FGFR1, FYN, GSK3β, HIPK1, IKKα, JAK1, KIT, MEK1, MET, MKK6, MSK1, mTOR/FKBP12, NEK1, p70S6K, PAK4, PDK1, PI3K(p110α/p65α), PIM1, PKCα, RSK2, SRC, TBK1, or TLK1 kinase and their respective substrates were incubated with or without Ethyl ferulate in an in vitro kinase assay. Data are shown as mean ± S.D of values. [file Image_1.jpeg]

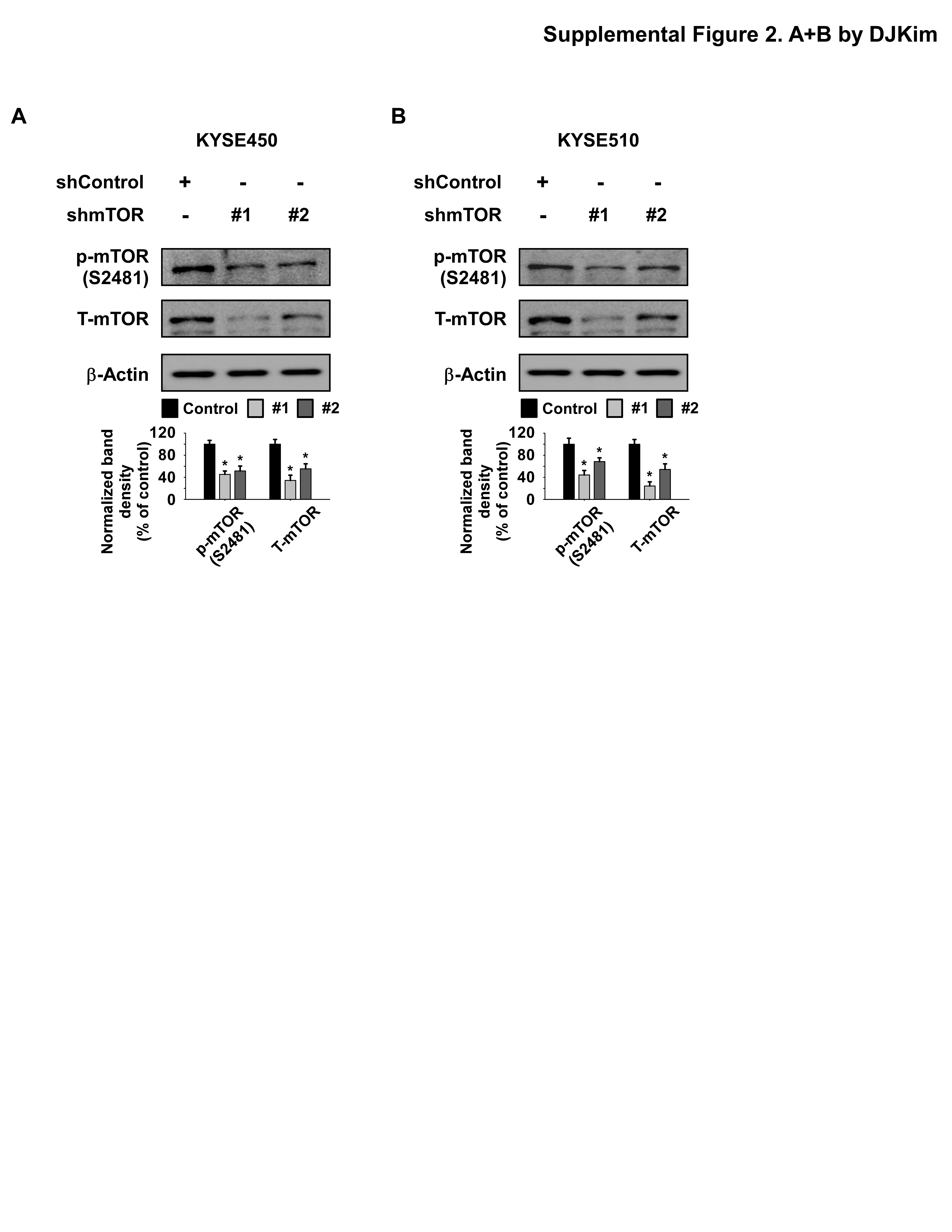

Supplement: Supplementary Figure 2 — Expression of mTOR protein in mTOR knockdown cells. (A, B) Effect of mTOR knockdown on total or phosphorylated mTOR protein expression in KYSE450 (A) and KYSE510 (B) cells. KYSE450 and KYSE510 cells stably expressing shRNA control or shmTOR were analyzed by Western blotting. Band density was measured using the Image J (NIH) software program. For all experiments, similar results were observed from 3 independent experiments and band density is shown as a bar graph. One-way ANOVA was used to compare significant differences. [file Image_2.jpeg]

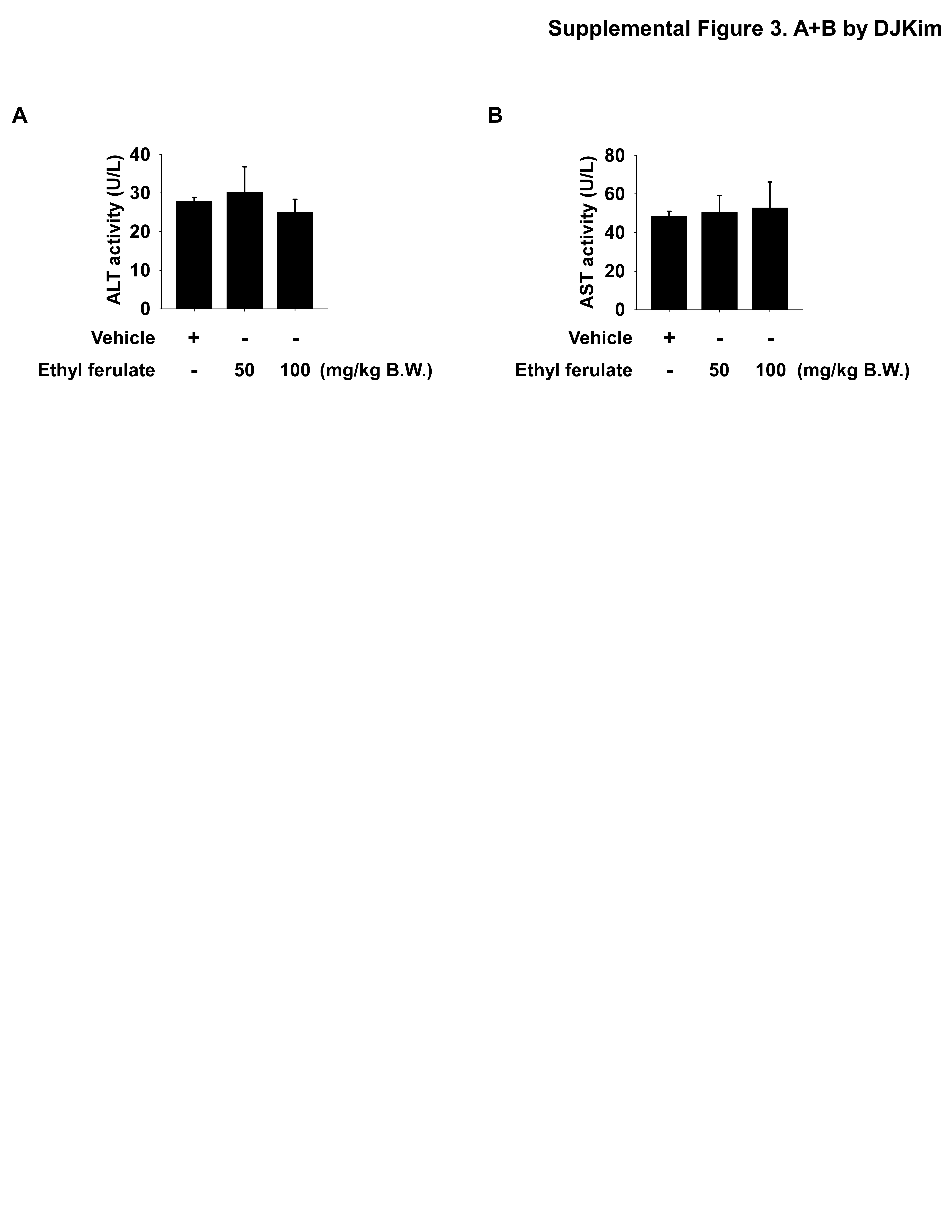

Supplement: Supplementary Figure 3 — Effect of Ethyl ferulate on ALT and AST activity. (A, B) The effect of Ethyl ferulate on ALT (A) and AST activity (B) was determined. Mice were orally administrated Ethyl ferulate at 50 mg/kg, 100 mg/kg, or vehicle once a day Monday through Friday for 2 weeks by the gavage method. Blood samples from mice were collected and analyzed. The ALT and AST activity were measured in mice treated with Ethyl ferulate and the vehicle-treated group. Data are shown as mean ± S.E. of values obtained from each group (n = 4). [file Image_3.jpeg]

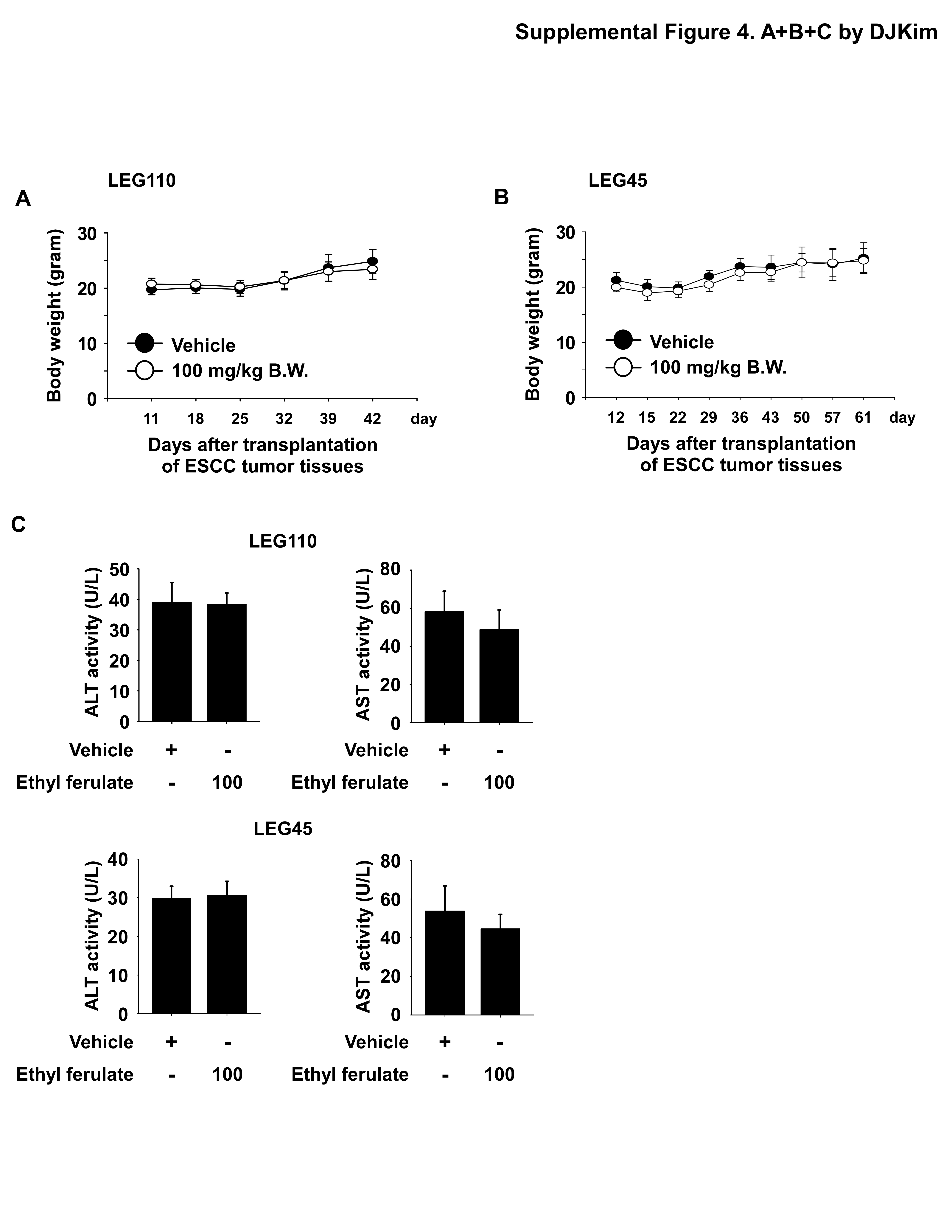

Supplement: Supplementary Figure 4 — Effect of Ethyl ferulate on mice bodyweight, ALT and AST activity. (A, B) Effect of Ethyl ferulate on mice body weight. Vehicle or Ethyl ferulate at 100mg/kg was administered to mice by oral gavage, once a day for 32 (LEG110 tissue) or 53 (LEG45 tissue) days. The body weights of mice were treated with Ethyl ferulate or vehicle were measured once a week over the timespan of 32 (LEG110 tissue) or 53 (LEG45 tissue) days. (C) Ethyl ferulate exhibits no obvious toxicity in vivo. The effect of Ethyl ferulate on the ALT and AST activity were determined. Blood samples from each group (n = 7) were collected and analyzed. The ALT and AST activity were measured in mice treated with Ethyl ferulate or vehicle. All data are shown as mean ± S.E. of values obtained from experiments. [file Image_4.jpeg]

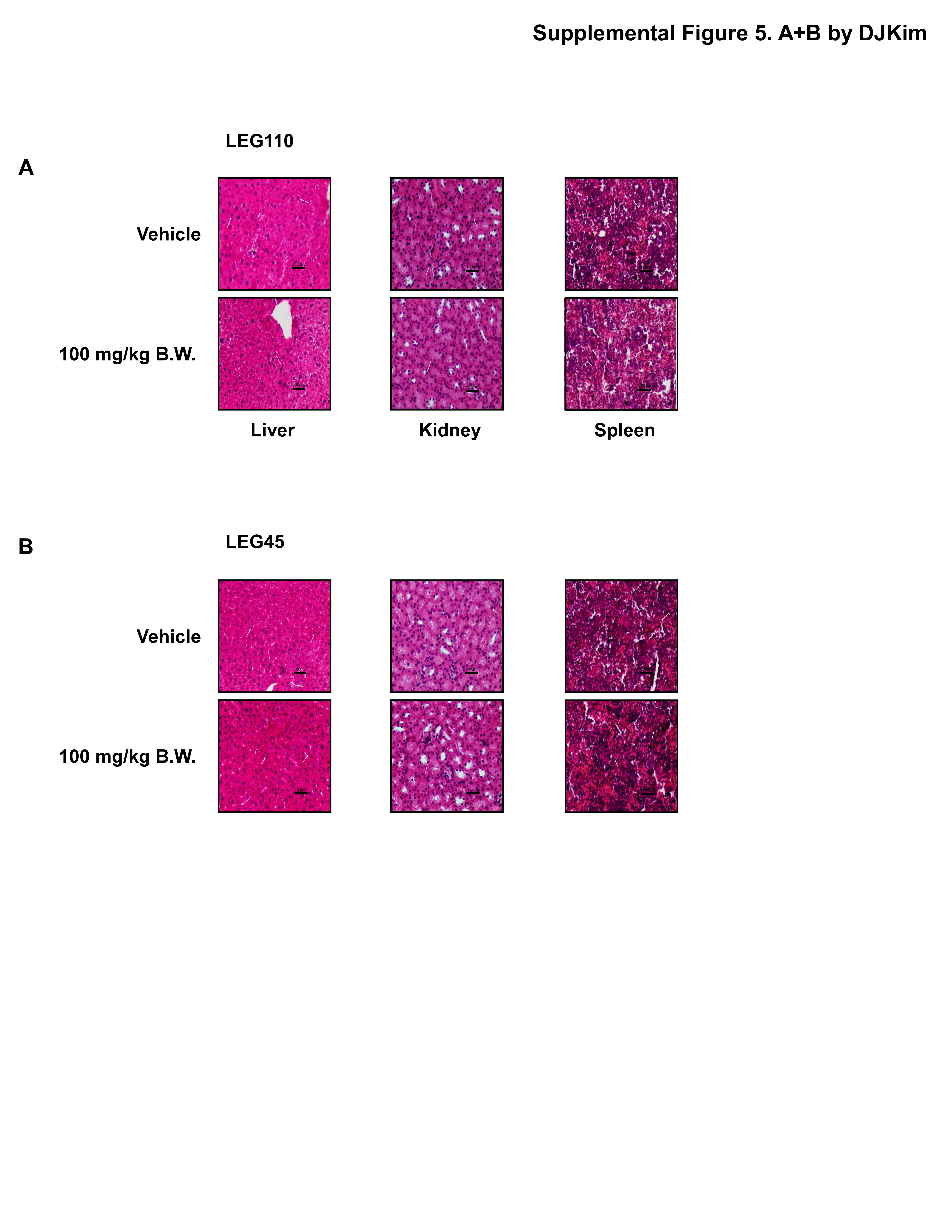

Supplement: Supplementary Figure 5 — Ethyl ferulate has no obvious toxicity in vivo. (A, B) H&E staining of liver, kidney, or spleen isolated from Ethyl ferulate-treated and untreated mouse groups inoculated with LEG110 (A; n = 5, 3 slides per tissue) and LEG45 (B; n = 5, 3 slides per tissue) tissues (100×; Scale bar: 100 μm). [file Image_5.jpeg]

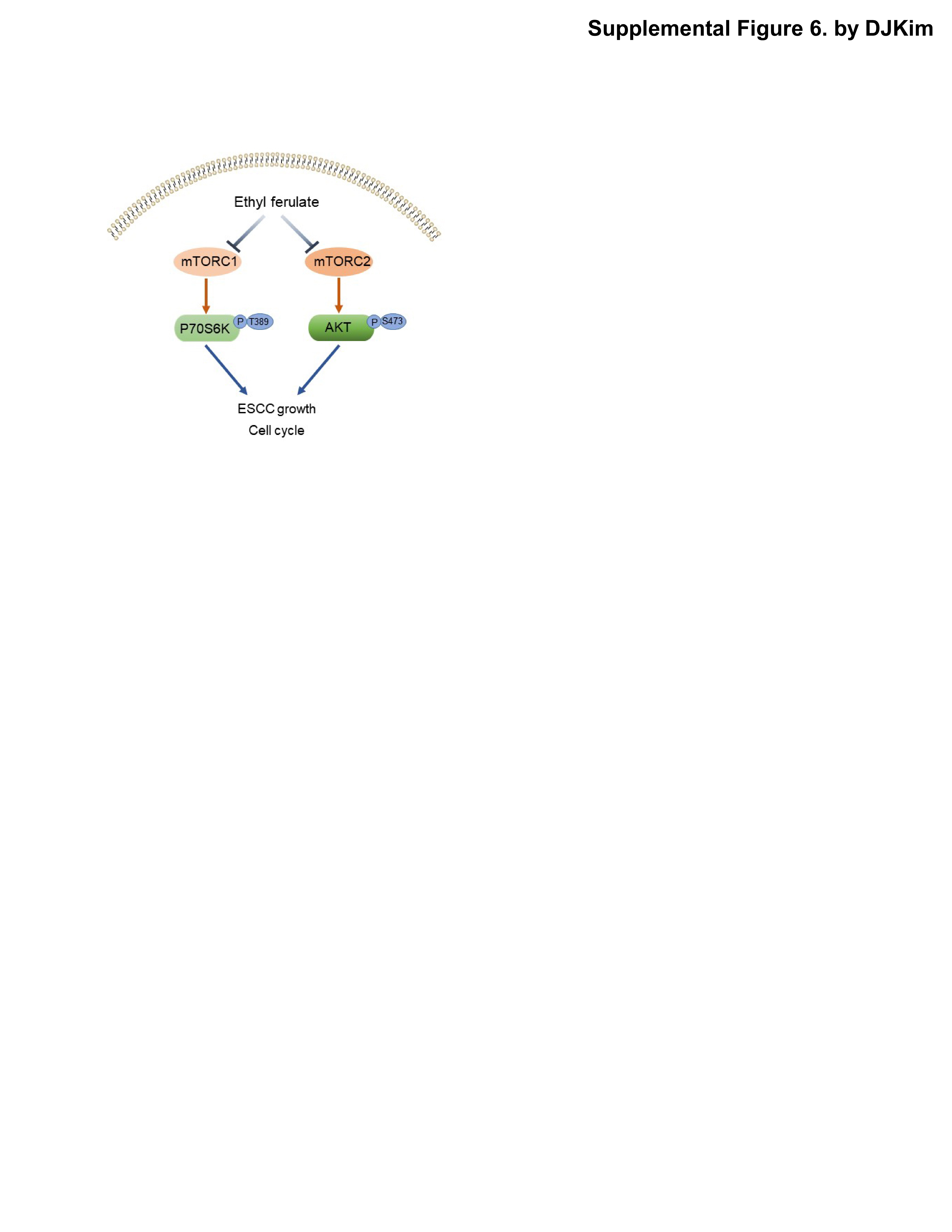

Supplement: Supplementary Figure 6 — Representative diagram of the mechanism of the anti-cancer activity of Ethyl ferulate. [file Image_6.jpeg]
